# Is Low Male Fertility Adaptive? Implications for Male Infertility Research

**DOI:** 10.1111/andr.70433

**Published:** 2026-11-09

**Authors:** John K. Amory

**Affiliations:** ^1^ Department of Medicine University of Washington Seattle Washington USA

**Keywords:** negative regulation, pregnancy, reproduction, sexual selection, spermatogenesis

## Abstract

**Background:**

Fertile human males produce significantly fewer sperm per gram of testes than most other animals, including primates. Human's low sperm output is associated with a lower pregnancy rate per episode of intercourse and a longer time to pregnancy than in most other species. This, coupled with the concealed nature of ovulation in women, means that human couples frequently require numerous acts of intercourse to achieve a pregnancy. The reason for the low fecundability of humans is unknown.

**Methods:**

I performed a literature review examining the efficiency of spermatogenesis and time to pregnancy in humans to estimate the period of time and average number of episodes of sexual intercourse required for pregnancy. These estimates were corroborated with mathematical models. Hypotheses are suggested to explain the low fecundity of humans.

**Results:**

The average, fertile human couple has sexual intercourse 17–35 times over 2–6 months to conceive, with some fertile couples requiring longer periods of time. These numbers and time period are greater than for most other species. Among the theories that could be advanced to explain the relatively low fecundability of humans compared with other species, two will be explored here: (i) a reduction in the risk of pre‐eclampsia with repeated sexual intercourse with a single partner over several months and/or (ii) improved pair‐bonding from repeated intercourse over time leading to improved outcomes for children.

**Conclusions:**

Reduced sperm production in men may be evolutionarily adaptive. Lower fertility and longer time to conception is associated with a reduced risk of pre‐eclampsia and improved outcomes for offspring. Greater understanding of the mechanism(s) involved in the relatively low sperm output in men might provide clues to explain some types of male infertility. Additional research into the reduced efficiency of sperm output in men is warranted.

## 1 Introduction

The human population currently exceeds eight billion and continues to increase by approximately sixty million yearly, although the growth rate has declined over the last 40 years and worldwide population is projected to stabilize or even drop slightly over the next century [1]. Notably, over the last 100 years, human population has quadrupled from a little over two billion to its current level as a result of major technological advances in medicine, public health and sanitation, and agriculture [2]. This dramatic increase in human population is all the more remarkable because humans have a lower capacity for reproduction, or fecundity, than most other species.

Given the concealed nature of ovulation in women, the chance of pregnancy from a single act of intercourse in a fertile human couple is approximately 3%, with the chance of conception varying between 0–1% during menses up to 20%–25% on day 14 of the menstrual cycle [3, 4, 5]. When multiple acts of intercourse are accounted for, couples have a 10%–30% chance of conceiving per menstrual cycle, depending on the frequency of intercourse [6, 7], with cumulative conception rates of 60%–75% at 6 months and 80%–90% after 1 year depending on the cohort [8, 9, 10]. What is evident from these data are that humans frequently require numerous acts of intercourse over a significant period of time to achieve a pregnancy. In contrast, the chance of pregnancy from a *single* act of intercourse in species that exhibit estrus as a physical manifestation of ovulation such as mice, rats, dogs, horses, pigs, sheep, cattle, deer, and many non‐human primates exceeds 50%. Even more impressively, in animals who exhibit sexually‐induced ovulation, such as cats, camels, rabbits and bears, the pregnancy rate after a single episode of intercourse can exceed 90% [11].

The reason for the low fecundity of humans compared to other species is unknown. Fertility is a function of several factors in both the male and female partner. In women, factors such as age, female ovulatory function, abnormal anatomy, and history of sexually transmitted infections are common reasons for reduced fertility and have received a great deal of attention. The most important mechanism limiting human fertility is the high rate of embryonic aneuploidy precipitated by chromosomal nondisjunction [12]. The risk of chromosomal non‐disjunction increases significantly with female but not male age [13]. This risk is possibly due to the long period of arrested prophase I of meiosis in the human female allowing for degradation of the meiotic spindle and a loss of cohesion between sister chromatids [14, 15].

Historically, less attention has been paid to the male contribution to fertility, despite the fact that 30%–50% of infertility is male in origin, and is often caused by reduced sperm output [16]. Indeed, to my knowledge, there has not been prior discussion in the literature about the male contribution to low human fertility relative to other species and its drivers.

In this short communication, I hypothesize that low human fertility may be evolutionarily adaptive. Low sperm output in men necessitates an increase in the number of episodes of sexual intercourse and time to achieve a pregnancy. Low sperm output in men may be paradoxically selected for over time to maximize reproductive fitness. This could be due, for example, to increasing the female partner's exposure to semen and thereby reducing the risk of pre‐eclampsia. Alternatively, it could be due to increasing pair‐bonding in the couple leading to improved childhood outcomes and reproductive success of the resulting offspring. These hypotheses, and their implications for understanding the regulation of male fertility and research into the causes of male infertility, are discussed.

## 2 Methods

I performed a literature review in PubMed in December 2025 examining the efficiency of spermatogenesis in men and animals, data from observational human time to pregnancy cohorts and prospective studies examining the chance of pregnancy in couples attempting a pregnancy. To understand the reasons for low efficiency of human spermatogenesis, I used the following search terms: “spermatogenesis”, “sperm efficiency”, “fecundity”, “fertility” “first pregnancy planning,” and “time to pregnancy”. Using reported values for the chance of pregnancy per cycle per act of sexual intercourse, mathematical estimates of the time in months or number of episodes of sexual intercourse needed by human couples to achieve a pregnancy were generated using the continuous distribution function of the binomial distribution as previously demonstrated [17]. This function estimates the cumulative chance of an event in “*n*” trials each with a known frequency of success. Using this function, the cumulative chance of pregnancy increases with each episode of sexual intercourse, starting at 3% with the first episode and increasing thereafter can be estimated. These calculations were based on data from a large prospective trial [4, 5, 18]. Lastly, to generate hypotheses as to the potential evolutionary benefit of reduced spermatogenesis and human fertility, literature search was performed using the following search terms: “pre‐eclampsia risk”, “miscarriage risk”, “maternal tolerance”, “childhood outcomes,” and “parental relationship”. All statistical modeling was performed using STATA version 10 (College Park, TX, USA).

## 3 Results

### 3.1 Human Spermatogenesis is Less Efficient Than in Other Species

Daily sperm production per gram of testicular parenchyma is a measure of the efficiency of spermatogenesis and can be useful for understanding interspecies differences in spermatogenesis [19]. Amann and Johnson have conclusively shown that humans have a much lower efficiency of spermatogenesis compared to other species [20, 21, 22, 23] (Figure 1). Most notably, the efficiency of spermatogenesis in humans is roughly 25% that of Rhesus monkeys. The lower efficiency of spermatogenesis in humans as compared to other species is thought to be a function of a longer duration of spermatogenesis and slightly lower percentage of the testes being occupied by seminiferous tubules [24], although these factors alone do not account for the markedly decreased efficiency of spermatogenesis in humans compared with other species and more research will be needed to understand the factors contributing to species differences in spermatogenic efficiency. Notably, there is a roughly 50% drop off in germ cell numbers in humans when comparing primary spermatocyte number with round spermatid number, suggesting a significant “checkpoint” occurs during meiosis in humans [19].

**FIGURE 1:**
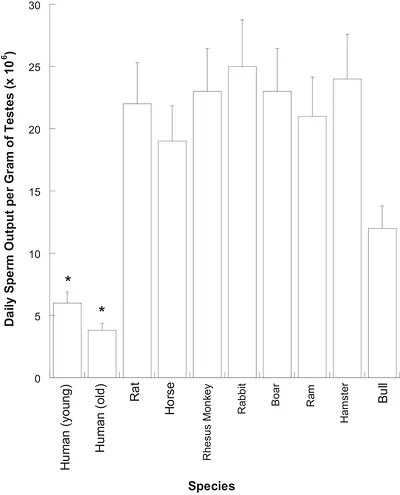
Efficiency of spermatogenesis in various species based on daily sperm production per gram of testicular parenchyma. Data are from research conducted by Dr. Larry Johnson and Dr. Richard Amelar and colleagues, see refs. [19–23] for details of methods and results. *<0.001 compared with all other species except bull and <0.01 compared with bull.

### 3.2 Average Time to Pregnancy in Humans is 2–6 Months

Time to pregnancy is a commonly used observational method to study human fertility. In time to pregnancy studies, women are interviewed about the duration of time from initiation of unprotected intercourse to conception [9 ^,^ 25]. Studies of this type can be used to examine for trends over time in human fertility and differences between locales or exposures. These studies consistently show an average time‐to‐pregnancy of 2–6 months in a variety of populations, with significant effect modification observed due to the woman's age and cycle length [8, 26, 27, 28, 29]. More recently, a study analysis over 8000 women using a fertility app for help with conception showed an average time to pregnancy of 3.9 months [30].

The observed chance of pregnancy from a single act of intercourse in a fertile human couple is approximately 3% [3, 4, 5], and when multiple acts of intercourse are accounted for, couples have a 10%–30% chance of conceiving per menstrual cycle [6, 7]. Using these likelihoods, I generated several mathematical models to estimate time to pregnancy based on a 10%–30% chance of conception per cycle (Figure 2A) and a 2%–4% chance of conception per act of sexual intercourse (Figure 2B). These models are congruent with the aforementioned time to pregnancy data in that the median time to pregnancy from these models is between 2–6 months and 17–35 episodes of sexual intercourse. Note that the mathematical models estimate that the likelihood of pregnancy over time in a couple is still quite high, with 80%–90% of couples conceiving after 12 months or approximately 100 episodes of sexual intercourse. These estimates are very similar to those observed in time to pregnancy studies [8, 26, 27, 28, 29].

**FIGURE 2:**
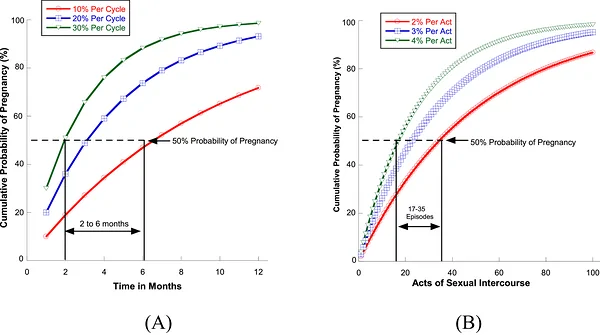
Mathematical models of the chance of pregnancy by month by probability of pregnancy per cycle of 10%, 20%, or 30% chance or pregnancy per cycle (A). Chance of pregnancy by number of acts of sexual intercourse by probability per act of 2%, 3%, or 4% chance of pregnancy per act (B).

### 3.3 Association of Longer Time to Pregnancy With Reduced Pre‐Eclampsia Risk

Well‐tuned modulation of the maternal immune response to promote tolerance of the fetus is critical for a successful pregnancy. At the maternal fetal interface, antigen‐presenting cells promote skewing of T‐cell differentiation toward regulatory phenotypes [31]. However, it has recently been shown that “priming” of the women's immune system starts prior to embryo implantation when resident immune cells in the genital tract encounter paternal antigens introduced by semen [32, 33, 34]. Several soluble cytokines are present in semen at physiologically relevant concentrations [35]. In addition, semen also contains copious quantities of extracellular vesicles that include various species of RNA and immunomodulatory proteins, and carry allogeneic major histocompatibility complex molecules, many of which would be expressed by any resulting fetus [36, 37, 38].

Pre‐eclampsia is a serious hypertensive state manifesting after 20 weeks of pregnancy due to deficient placental implantation. Pre‐eclampsia is surprisingly common, occurring in 3%–10% of pregnancies. While the etiology of pre‐eclampsia is multifactorial and extremely complex, the risk of pre‐eclampsia is affected by both the genetics and the duration of the relationship with the male partner. For example, the risk of pre‐eclampsia is higher in nulliparous women and in women with less than 12 months of cohabitation with their partner [39]. One recent study examined the relationship of vaginal exposure to seminal fluid and the risk of pre‐eclampsia by factoring in the type of contraceptive used by the couple, with condoms minimizing semen exposure. In this case–control study of 258 cases of pre‐eclampsia, there was a significant association dose–response between greater semen exposure in the vagina and a lower risk of pre‐eclampsia [40]. Another recent observational study found a significant 77% risk of pre‐eclampsia in women using donor versus partner sperm for intrauterine insemination, without an increase in the risk of low birth weight or pre‐term birth [41]. Prospective trials of immune modulation via vaginal exposure to paternal antigens in woman at high‐risk for pre‐eclampsia have not been performed, so it is unknown if this association is causal. Nevertheless, it seems possible that increased exposure to a specific male partner's semen may decrease the risk of pre‐eclampsia. Notably, this “tolerizing” strategy may allow for wider variety in HLA/MHC antigens in offspring than would be possible without repeated seminal priming. Such a diversity in these immune molecules could be associated with increased immunity to a wide array of pathogens, a distinct survival advantage.

### 3.4 Concealed Ovulation and Paternal Investment

The occult nature of ovulation in women is fairly unique compared with most other species as it increases the time to pregnancy. This is because roughly 75% of randomly timed acts of intercourse will fall outside the pre‐ovulatory fertile window. So why do woman have concealed ovulation given its attendant reduction in fertility? The leading theories to explain concealed ovulation in women is the “paternal investment hypothesis” first suggested by Alexander and Noonan in 1979 [42]. Simply put, this hypothesis suggests that concealed ovulation in women evolved to increase human pair bonding due to the need for significant paternal investment in human offspring. Concealed ovulation in women led to a change in men's mating strategy. Rather than mating with multiple women in the hope that some of them were at the fertile point of their cycle, men instead chose to mate with a particular woman repeatedly throughout her menstrual cycle. Thus, frequent mating, necessitated by concealed ovulation, would be most evolutionarily successful [43]. In addition, the additional sexual episodes would serve to enhance conjugal love and communication between partners fostering greater pair bonding [44]. Such a sustained pair bond would likely be advantageous throughout the period of pregnancy, lactation and rearing of the offspring as these require significant time and energy from the woman, while her own ability to forage is reduced. The offspring benefits from the pair‐bonded male in the form of additional food and defense. According to this theory, through this shared parental investment, both the man and the woman increase their offspring's chance for survival [45]. In this context, reduced spermatogenesis could also contribute to greater pair bonding by ensuring that pregnancy is not immediate and requires several acts of sexual intercourse within a male–female prospective parent dyad.

### 3.5 Implications of Pair Bonding on Childhood Outcomes

There is an extensive observational literature demonstrating that children who grow up with two parents have better outcomes on average than children who grow up with a single parent. Preferential outcomes for children with two parents are apparent prenatally with unmarried, single and cohabitating mothers exhibiting significantly increased risk of low birth weight, pre‐term birth and small for gestational age infants compared to married mothers [46]. This advantage is also present across multiple health domains, with children in single‐parent households having two‐ to five‐fold higher risks of psychiatric disease, substance abuse and all‐cause mortality compared to children in two‐parent households, even when controlled for socioeconomic status [47, 48]. Growing up with two parents also protects against poverty and nutritional deprivation in early childhood and is protective against obesity in the longer‐term [49]. Fortunately, most children in single‐parent homes do well, but there are significant advantages of two‐parent households in observational studies, consistent with the overall notion that low fertility in humans allows for greater pair bonding prior to conception and therefore improved fitness of the resulting offspring. This discussion must acknowledge that the contemporary data supporting two‐parent families are from a very different economic context than the evolutionary pressures that shaped human reproductive fitness and it cannot be assumed that this would have applied throughout evolutionary history.

### 3.6 Known Factors Increasing and Decreasing Spermatogenesis in Humans

Spermatogenesis and sperm output are impacted by myriad factors, including anatomic, hormonal, toxic, genetic and nutritional inputs. For example, dozens of mutations have been identified that are associated with severe forms of male infertility [50]. Most of the known mutations causing male infertility are associated with very severe reductions in spermatogenesis. Yet, taken together, known genetic mutations and congenital conditions only account for ∼40% of cases of male infertility, meaning that in a majority of cases a given man's infertility is idiopathic [16]. Due to this, more recent research focused on epigenetic and proteomic approaches to understanding male infertility has been performed. For example, Greither and colleagues performed high‐resolution analysis of the sperm proteome and found over 7000 coding genes in the human sperm proteome [51]. Forty‐eight proteins with reduced abundance in men with infertility were identified, including some not previously described. Interestingly, only one protein was *upregulated* in infertile men in their sample, the apoptosis trigger APAF1. This protein, the apoptotic protease activating factor 1, is a crucial protein in the intrinsic pathway of programmed cell death, as it acts as the central scaffold of the apoptosome, the complex that activates caspase‐9, leading to cell suicide. This protein was more than three‐fold elevated in sperm from men with infertility, but is also present in the normal testes, suggesting a significant degree of apoptosis is present even in the sperm of men with normal fertility. It therefore seems possible that human spermatogenesis uses apoptosis or other similar mechanisms to reduce sperm output and hence fertility for the reasons noted above. Indeed, laboratory testing frequently reveals that up to 20% of sperm in the human ejaculate are undergoing apoptosis [52], a figure higher than most other species studied [53]. Somewhat paradoxically, such negative regulation of spermatogenesis may maximize human reproductive fitness over time by reducing the risk of pre‐eclampsia and/or improving male–female pair bonding and subsequently the reproductive fitness of the resulting offspring. However, if excessive, negative regulation of sperm output could play an etiological role in some types of male infertility. Certainly, additional genetic, epigenetic and proteomic work will be required to fully understand the cause of infertility in the 50%–60% of men whose infertility remains idiopathic. An alternative mechanism to explain the low efficiency of human spermatogenesis compared to other animals could be a lack of selection pressure mediated by human mating strategies. Specifically, the largely monogamous nature of human reproduction may allow for sperm parameters such as concentration, motility and morphology that are simply sufficient for conception in the context of mutual monogamous matings. For example, this lack of sperm competition is observed in gorillas in contrast to promiscuous chimpanzees, in who sperm competition likely has led to larger testes and greater sperm production [54].

## 4 Discussion

Evolutionary biologist Graham Bell called sexual reproduction “the masterpiece of nature” [55]. In his 1982 book of the same name, he examined why sexual reproduction persists despite the “cost of sex,” which specifically refers to the fact that sexual reproduction leads to offspring who have only half of the genes of the parent. Sexual reproduction in humans is particularly costly when the relatively high risk of death from pre‐eclampsia and complications of childbirth for women and the outlay of time and resources required to support human children to independence is considered. Therefore, it is likely that mechanisms to maximize the fitness of human offspring have evolved in tandem with the particular reproductive challenges of *Homo sapiens*, the “wise apes.”

One such mechanism well described in the evolutionary biology literature is the concealment of ovulation in women. As noted above, the most widely accepted theory is that concealed ovulation led to increased paternal involvement and greater pair bonding in couples thereby maximizing the fitness of the resulting offspring. Concealed ovulation in females makes it more likely that multiple episodes of sexual intercourse will be required for a conception to occur. Based both on data from large prospective trials and mathematical models, I have estimated that the average fertile human couple will need to have sexual intercourse 17–35 times over 2–6 months to result in a pregnancy, long enough to increase the probability that the couple is pair bonded before a pregnancy can occur.

In this short communication, I suggest that the low efficiency of sperm production in men also contributes to human's relatively long time to pregnancy period. Reduced efficiency of sperm production in men may contribute to both a reduction in the risk of pre‐eclampsia with increasing duration of partnership via protracted exposure to paternal antigens and/or improved pair‐bonding from repeated intercourse leading to improved outcomes for the conceived children. Because these associations are based on observational data, causality is unproven and this hypothesis remains speculative. Notably, the relative delay in human fertility appears not to significantly decrease long‐term fertility as 85% of couples successfully become pregnant in 6–12 months of attempting to conceive [8, 9, 10]. Ultimately, human fertility is influenced by multiple known factors, including female reproductive physiology, behavioral patterns and social and environmental conditions. The discussion presented in this short communication is intended to also examine the male contribution via relatively inefficient spermatogenesis to our low fecundability compared to most other species.

Any adaptive reduction in human fecundity mediated through spermatogenesis necessarily implies negative regulation of spermatogenesis via some mechanism, the most likely of which is apoptosis. If this negative suppression were exaggerated, it could lead to male infertility due to excessive apoptosis and poor sperm output in some men. What could be suppressing sperm output in men? At present, this is unknown. However, a theoretical possibility could include “spermatogenesis suppressor” gene(s). “If such a spermatogenesis suppressor” gene was upregulated (e.g., by a copy number increase), or a second gene or epigenetic or environmental factor that down‐regulated the spermatogenesis suppressor gene were impaired, excessive suppression of spermatogenesis leading to male infertility could result. Such mechanisms might help to explain male infertility in some of the 60% of men with decreased sperm output in whom clear genetic, anatomic, toxic, congenital or other causes have not been identified.

In conclusion, in this short communication I have hypothesized that the markedly reduced efficiency or sperm production in men compared with other species may be evolutionarily adaptive and associated with a longer time to conception and a reduced risk of pre‐eclampsia and/or improved outcomes for offspring. Regardless of the cause, an adaptive reduction in sperm output would require mechanism(s) to suppress spermatogenesis, which could be a heretofore undescribed etiology of male infertility. Additional research into suppressive mechanisms that limit sperm production in both normal and infertile men is warranted.

## Author Contribution

Dr. John K. Amory conceived of and wrote this manuscript.

## Funding

Dr. Amory receives research funds from Celldex Therapeutics and NEXT Life Sciences.

## Data Availability

Data sharing not applicable to this article as no datasets were generated or analysed during the current study.
